# Cocoa bean fingerprinting via correlation networks

**DOI:** 10.1038/s41538-021-00120-4

**Published:** 2022-01-24

**Authors:** Santhust Kumar, Roy N. D’Souza, Marcello Corno, Matthias S. Ullrich, Nikolai Kuhnert, Marc-Thorsten Hütt

**Affiliations:** 1 Department of Life Sciences and Chemistry, Jacobs University Bremen, Campus Ring 1, 28759 Bremen, Germany; 2 Barry Callebaut AG, Westpark, Pfingstweidstrasse 60, Zurich, 8005 Switzerland

**Keywords:** Metabolomics, Mass spectrometry

## Abstract

Cocoa products have a remarkable chemical and sensory complexity. However, in contrast to other fermentation processes in the food industry, cocoa bean fermentation is left essentially uncontrolled and is devoid of standardization. Questions of food authenticity and food quality are hence particularly challenging for cocoa. Here we provide an illustration how network science can support food fingerprinting and food authenticity research. Using a large dataset of 140 cocoa samples comprising three cocoa fermentation/processing stages and eight countries, we obtain correlation networks between the cocoa samples by computing measures of pairwise correlation from their liquid chromatography-mass spectrometry (LC-MS) profiles. We find that the topology of correlation networks derived from untargeted LC-MS profiles is indicative of the fermentation and processing stage as well as the origin country of cocoa samples. Progressively increasing the correlation threshold firstly reveals network clusters based on processing stage and later country-based clusters. We present both, qualitative and quantitative evidence through network visualization, network statistics and concepts from machine learning. In our view, this network-based approach for classifying mass spectrometry data has broad applicability beyond cocoa.

## Introduction

Food fingerprinting has been discussed from a multitude of technological perspectives^1–3^. However, the sophistication of the analytical instruments is often in contrast to the lack of innovation on the computational and data analysis side. Here we illustrate – using a large data set of cocoa liquid chromatography coupled to mass spectroscopy (LC-MS) data from eight countries of origin and in three processing stages (unfermented, fermented, liquor) – how a network-based analysis can reveal the underlying patterns, embedded in interconnected biochemical and chemical reaction grids.

Such a characterization of cocoa samples in terms of processing stages and country of origin is a prerequisite for quality control and fine flavor products^4,5^. Traditional approaches (e.g., PCA) can successfully reveal groups of unfermented and fermented cocoa samples^6,7^. However, especially when the number of countries in the dataset is not small, grouping of samples on the basis of countries of origin has remained elusive^6,8,9^.

In its most basic form, a network-based interpretation of high-throughput or alternative complex data offers a dimensionality reduction of the raw data, thus dramatically facilitating further data analysis steps and increasing the statistical significance of results^10,11^. This basic idea has revolutionized the way we analyze diverse biological and medical data and serves as a foundation of what is called today Systems Biology and Systems Medicine^12–14^. In most applications, this dimensionality reduction is achieved using a given biological network (e.g., a metabolic network or a protein-protein-interaction network), which is in turn a condensed representation of a vast inventory of biological knowledge^15–17^. As pointed out by Bartel et al.^18^, when no external network is available or in order to reduce the uncertainty due the unreliability or the incompleteness of the given biological network, one can also resort to the intrinsic network contained in the data itself, spanned by the correlations among the different dynamical units. For a long time, this correlation network approach to analyze high-throughput data has been a prominent approach in metabolomics^19–28^. The approach has also found applications, refinements and development in the field of finance and economics^29,30^.

Efforts of a fundamentally new perspective on complex systems are now often summarized under the term ‘network science’^31,32^. Network science operates under the assumption that mathematical graphs–objects consisting of nodes and links–serve as suitable abstractions of real-life systems: retaining enough detail to be informative but generalizing enough to allow for the application of a standardized set of tools and for inter-system comparisons.

The main purpose of our investigation is to bring this field of network science to food research. Specifically, we quantify classifiability of cocoa mass spectrometry data due to the network-based dimensionality reduction.

Cocoa products have enormous flavor complexity stemming from numerous factors, starting right from its genotype, weather conditions, harvesting, fermentation, drying, to its intermediate and final processing in a factory^33^. Various classes of chemical compounds, e.g., alkaloids, carbohydrates, polyphenols, peptides react with each other during processing to form a myriad of sensory precursors and ultimately taste and aroma active compounds such as aldehydes, ketones, pyrazines, diketopiperazines, etc., contributing to its unique flavor. Metabolomics analysis through techniques like LC-MS offer a high throughput quantitative access to this complexity.

Chemometrics, the broad discipline of applying mathematics and statistics to Chemistry, offers a range of tools for interpreting such datasets, but so far the underlying rules, how key features of cocoa harvest, fermentation and production shape these LC-MS data, have remained elusive^34,35^. The limitations of common statistical approaches, like PCA become apparent, when going back to the formal definition of these methods^36^. Technically, PCA searches for the linear combinations of components of the high-dimensional data set, along which variance across samples is maximal. Consequently, PCA leads to meaningful subcategories, if the (true) subcategories are the main drivers of variation among samples. This is particularly striking, when we are setting out to identifying multiple types of subcategories in the same set of samples (e.g., countries of origin, fermentation status or stage of industrial treatment in the case of the cocoa samples discussed here;^6^ or variety and geographical origin in case of wines;^37^ or farming practices and cultivation region in case of coffee;^38^ roasting and geographic origin in case of coffee^39^). Often however, the patterns of variances among samples arise from an overlay of contributions from multiple sources, by no means limited to the parcellation of samples into the subcategories of relevance to food industry. These limitations of PCA are, in part, responsible for its lack of success in food fingerprinting (e.g., the classification of cocoa samples into origin countries based on their LC-MS profile^6,8,9,40–42^).

**Fig. 1:**
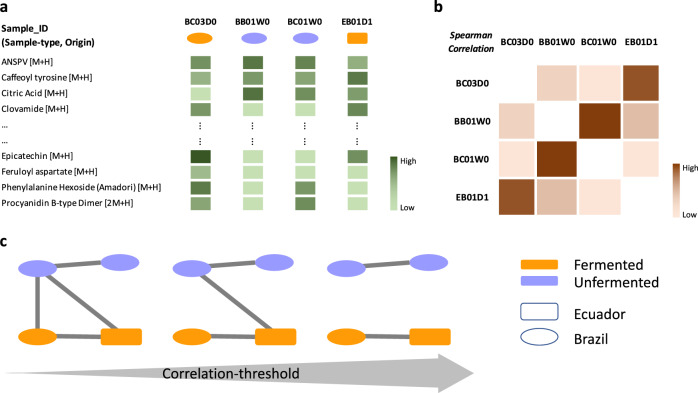
Schematic illustration of working procedure. (**a**) Schematic of LC-MS data structure. Subset of real LC-MS dataset (compounds in rows, samples in columns): the darker the color of a box, the higher the concentration of the compound in the sample. In this schematic illustration a couple of samples from Ecuador and Brazil of unfermented and fermented categories are shown. (**b**) Schematic representation of correlation matrix. Spearman correlation between different samples. (**c**) Schematic of network generation. Correlation networks as a function of increasing correlation thresholds. An edge exists between two nodes only when the correlation between them is greater than or equal to some specified threshold.

In a combination of chemometrics and network science we derive a set of correlation networks, parameterized by the correlation threshold defining a link, from 140 cocoa samples belonging to three different stages in a typical cocoa processing pipeline (unfermented, fermented and liquor) and 8 countries through their LC-MS profiles in positive ion mode. A schematic overview of the generation of correlation networks out of the LC-MS data is provided in Fig. 1.

We find that, as we progressively increase the correlation threshold from 0 towards 1, the clustering of cocoa samples is first dominated by processing-stage sample types at low and intermediate correlation thresholds, and then by countries of origin at high correlation thresholds. We show this both qualitatively and quantitatively via network visualizations, network edge statistics and other indicators of classifiability. This family of correlation networks, a common tool from early metabolomics research^19,26^, is in this way here linked to the notion of classification accuracy commonly applied in machine learning.

Our work demonstrates the presence of processing-stage level grouping on a coarser level and origin level grouping on a finer level within the former. This nested grouping can be revealed by successively keeping higher correlations. Varying the correlation threshold, the functional hierarchy of structures in this family of networks is thus revealed.

## Results

### Processing stage modules: block-structures in correlation matrix

**Fig. 2:**
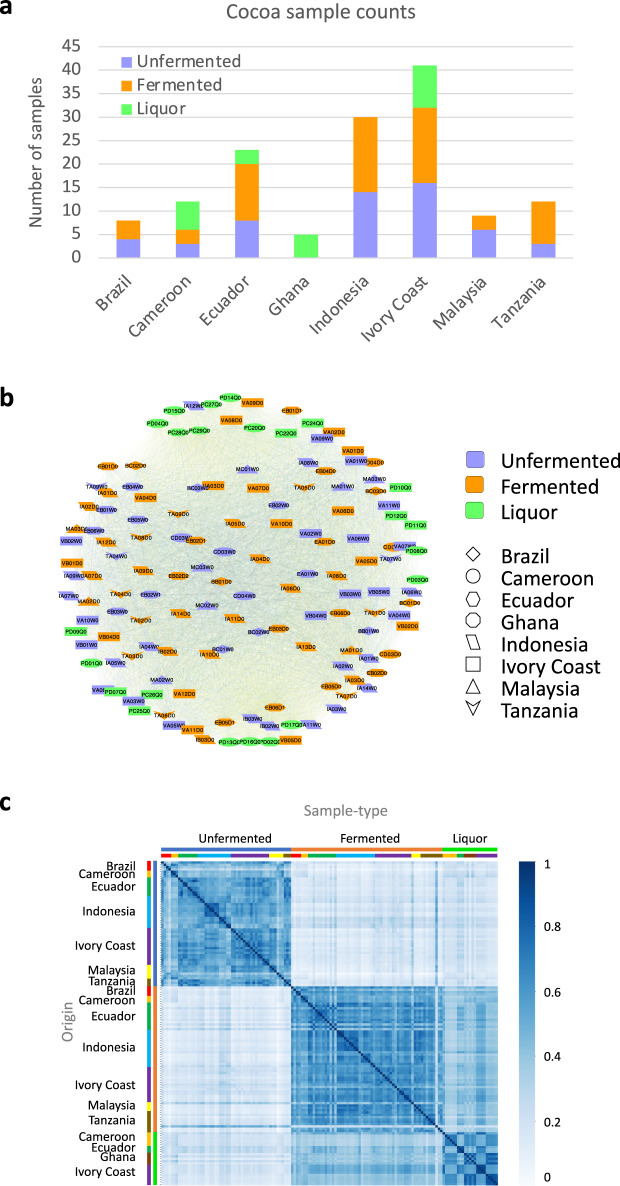
Initial network details. (**a**) Distribution of Cocoa samples used in this study—a total of eight countries and three cocoa processing stages are represented. Ivory Coast contributes most samples and Ghana the least. (**b**) Correlation Network. Full correlation network made using all correlations between the set of cocoa samples using Spearman correlation (i.e., at correlation threshold of zero). The nodes are color coded according to their processing-stage sample type and shape coded by their country of origin. The network is visualized using Cytoscape^52^ with ‘edge-weighted spring embedded layout’ which keeps nodes connected with higher correlations closer together. (**c**) Correlation Heatmap. Darker regions represent high correlation, and lighter regions represent low correlation. Samples have been sorted on twin axes, first on processing stage sample-type, and then second internally on country of origin. Two distinct square block regions are clearly visible along the diagonal of the matrix, corresponding to Unfermented (smaller block) and Fermented (bigger block) samples.

The Spearman correlation matrix ${{{\tilde{\mathrm R}}}}$ obtained this way is visualized through heatmap in Fig. 2c. (For the case of Pearson correlation coefficient matrix, R, see Supplementary Fig. 1) By definition, the correlation matrices ${{{\tilde{\mathrm R}}}}$ and R are symmetric. The twin attributes of nodes, namely the processing-stage sample type and country of origin, have been alternatively marked on the sides. Three blocks corresponding to Unfermented, Fermented and Liquor samples are clearly distinguishable. It is also visible that Fermented and Liquor samples are part of a larger block, which is separated from Unfermented samples. This shows that Liquor samples are closer in character to Fermented samples. This is in consonance with general expectation that liquor production follows the fermentation stage and that usually only Fermented beans are subject to Liquor formation. Furthermore, more chemical changes occur in cocoa when moving from unfermented stage to fermented stage than those occurring from fermented to liquor stage suggesting a greater impact of fermentation on generation of desirable cocoa features than during liquor formation. In case of correlation heatmap obtained using Pearson correlation (Supplementary Fig. 1) the block of Unfermented samples is clearly distinguishable from Fermented and Liquor samples, while the Fermented and Liquor samples are mildly distinguishable. Further, it is important to note that no block structure on the basis of country is discernable at this level of detail about the correlations.

Next, we define correlation network using the Spearman ($\tilde r$) and Pearson correlations (*r*) obtained above.

### Networks at low and intermediate correlation thresholds reveals processing-stage sample type modules

**Fig. 3:**
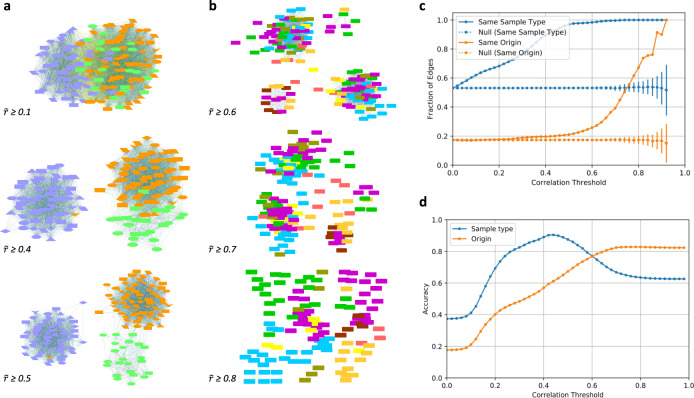
Network transformation as a function of varying correlation threshold. (**a**) Processing-stage modules: modules of LC-MS samples belonging to the same cocoa processing-stage in a typical cocoa processing pipeline. (${{{\tilde{\boldsymbol r}}}} \ge 0.1$) Mild separation of unfermented, fermented and liquor cluster; (${{{\tilde{\boldsymbol r}}}} \ge 0.4$) modular structure improves; (${{{\tilde{\boldsymbol r}}}} \ge 0.5$) groups of unfermented, fermented and liquor samples are clearly separated. The figure follows same legend as of Fig. 2b. See Supplementary Fig. 2 for a detailed version of networks and for a movie of evolving network as the correlation threshold is progressively increased. (**b**) Country modules: correlation thresholds of 0.6, 0.7 and 0.8. Several modules with nodes belonging to the same country of origin are revealed. For a quick and better comprehension, and unlike the legend of earlier correlation networks, in this figure, different countries are represented through a different color. (For corresponding node-labeled network see Supplementary Fig. 4. The networks with same thresholds but with previous node color/shape scheme is given in Supplementary Fig. 5) (**c**) Connected nodes’ similarity. The sample-type similarity (blue line) starts to increase linearly right from smaller correlation threshold values, reaches close to 1 around a correlation threshold value of 0.5. The origin similarity remains constant for a long range of correlation threshold (0, 0.50) and then increases rapidly. The dashed lines and error bars show corresponding similarities and standard deviation, respectively (see Similarity of nodes connected by an edge), as expected from an ensemble of control networks. (**d**) Accuracy of links in thresholded correlation networks, or closeness of a thresholded correlation network to expected ideal network. As the correlation threshold increases the threshold networks become closer to their ideal counterparts. (For an explanation of ‘accuracy’ through a toy-example, see Supplementary Fig. 10) In regions of lower correlation threshold, the thresholded networks describe the sample type character of the network more than the origin type character. In regions of higher correlation threshold, opposite is true and the thresholded networks are closer in their character to the origin attribute of LC-MS samples. This is coherent with the network pictures at various threshold seen in earlier figures.

We analyze correlation networks at low and intermediate correlation thresholds ($\tilde r$_*th*_), varying it from $\tilde r$_*th*_ = 0.1 to 0.5, in steps of 0.1. The network at a given correlation threshold contains all the edges with correlation greater than or equal to the set threshold. Some of these networks are visualized in Fig. 3a and show the networks at correlation thresholds of 0.1, 0.4, and 0.5, respectively. First, the nodes belonging to Unfermented samples are seen little separated from the nodes belonging to the Fermented and Liquor samples. Next, the Unfermented samples are clearly separated from the Fermented and Liquor samples. Within the Fermented and Liquor samples little grouping starts to form. Finally, all the three samples can be seen clearly separated from one another. This separation of samples first into two groups: (a) Unfermented, and (b) Fermented and Liquor samples, and then slowly into three groups: Unfermented, Fermented and Liquor samples, is in congruence with the earlier result seen in the structure of the correlation matrix heatmap shown in Fig. 2b. Both Fig. 2b and Fig. 3a show that the liquor sample are more similar to the fermented samples than to the unfermented samples. This is in accordance with the fact that major chemical and physical changes in cocoa beans takes place during the processes of fermentation. (For labeled nodes see Supplementary Fig. 2) A movie of the network as a function of progressively increasing threshold is attached as Supplementary Video 1 which shows the evolving network at intermediate correlation thresholds and the separation of samples belonging to different cocoa processing stage. Similar behavior is noted for the case of correlation network formed using the Pearson correlation coefficient (Supplementary Fig. 3) however at different values of correlation threshold.

### Country of origin-enriched modules at high correlation thresholds

As the correlation threshold is further increased, the network breaks into various smaller connected components. The resulting individual connected components primarily have the processing-stage sample type. However, there are more than one component that belong to same color or sample type. This reveals the internal structure of the clusters of samples that initially grouped on the basis of their sample types. This additional sub-structure of the network reveals grouping which now is primarily governed by the samples belonging to same country of origin. This is shown in the networks in Fig. 3b for correlation thresholds of 0.6, 0.7 and 0.8. In contrast to the legend used in previous figures, we now color the nodes on the basis of countries for a quick comprehension of grouping on the basis of countries. (The figure with the previous legend scheme is given as Supplementary Fig. 5) It can be seen from Fig. 3b that same color nodes tend to be present closer together. This feature is visible more in modules of smaller size, but it is also discernible in larger sized modules. We see that as the correlation threshold is further increased, most of the larger size modules break into smaller module, where nodes belonging to the same origin country are increasingly often connected. It should be noted that processing-stage and country of origin are the only major governing factors, on which grouping of samples is based. Other factors such as variety of cocoa hybrid, harvest season, geographical location and landscape of farm in the country can begin to play an important role with increasing correlation threshold. Hence the clustering is not perfect. The other governing factors can potentially lead to finer sub-modular structures in the network. This situation is more likely to be evident at still higher correlation thresholds.

As the correlation threshold is gradually increased, edges with correlation less than the threshold value are lost from the network. On one hand this leads to increased consideration of the edges with higher correlation in the determination of the layout of the network, while on the other hand this, naturally, leads to decrease in the number of edges, and, as isolated nodes are dropped, can also decrease the number of nodes in the resulting network, resulting in network breakage. The variation of number of edges and nodes connecting them is shown in the Supplementary Fig. 8. A movie of the network as a function of progressively increasing the threshold is attached as Supplementary Video 2 which clearly shows the evolving network and separation of samples belonging to different countries.

### Similarity of nodes connected by an edge

As every node in our correlation networks has two attributes, namely the processing-stage sample-type and origin, we define two kinds of similarity for a pair of nodes connected by an edge: sample-type similarity and origin similarity. We define sample-type similarity as the fraction of edges in a network connecting nodes having the same processing-stage sample-type attribute, and origin similarity as the fraction of edges in a network connecting nodes which have same origin attribute. For the sake of enhancing clarity and avoiding potential confusion, we would like to emphasize that edge-similarity is defined on edge basis and not over clusters observed in network representation at different thresholds.

The sample-type and origin similarities as a function of correlation thresholds based on Spearman correlation networks are shown in Fig. 3c (solid lines). They differ significantly from each other in terms of both the correlation threshold around which they start to rise and the manner in which they rise. The sample-type similarity starts to increase right from the smaller values of correlation thresholds itself and in a linear manner until it starts to saturate around a correlation threshold value of 0.5 to a similarity value close to 1. This is in agreement with the observed enhancement of the processing-stage sample type character of the network architecture right from the beginning of starting values of correlation threshold, to the almost full appearance of processing-stage sample type character at intermediate correlation threshold in large and small connected components (cf. panel A). The origin similarity remains almost constant and close to that of null model networks (orange dashed line) for a long range of correlation threshold (up to 0.5) suggesting a weak or almost negligible role in the clustering of nodes belonging to the same origin in the layout of network. Only when the correlation threshold is around 0.5, origin similarity starts to increase, suggesting this is the value of correlation threshold at which the contribution of origin effects start to contribute to clustering of nodes belonging to same origin. This clearly shows that the processing-stage sample type effect precedes the country effects, and the country effects are finer (require higher correlations) than the sample-type effect. The origin similarity increases rapidly and reaches a value close to 1 which implies that at higher threshold almost all edges connect nodes having same sample type and same country of origin.

The dashed lines along with error bars show similarity values and standard deviation expected from an ensemble of null model networks (control networks) obtained by randomizing edge weights in the original network (see Null model network or control network). The difference between the similarity values from original network and that obtained from null model networks point to the fact that the networks at higher correlation thresholds are enriched in edges that have high sample-type and origin similarity. The result corresponding to correlation network generated using Pearson correlation coefficient is given in Supplementary Fig. 9. Both show similar behavior, although at slightly different correlation threshold value.

### Closeness of threshold-determined networks to ideal networks

In this section, we quantify as a function of correlation threshold how accurately our networks represent the expected ideal networks of cocoa samples given their processing-stage sample types or country of origin. We consider two ideal networks, one each for the processing-stage sample type and country of origin. An ideal processing-stage sample type based network will have a link between a pair of its nodes only when both the nodes belong to the same processing-stage sample type, otherwise the link would be absent. Similarly, an ideal origin-based network will have a link between a pair of its nodes only when both the nodes belong to the same country of origin. Thus, in an ideal network based upon processing-stage sample type or country of origin a link is present only between nodes belonging to same sample type, or nodes belonging to same origin, otherwise there is no link between dissimilar nodes. After defining these ideal or true networks, we identify ‘true positive’ and ‘true negative’ links by comparing the links in the original network at a given correlation threshold (or threshold-determined network, for short) with the links in the ideal networks. A link is counted to be ‘true positive’ when the link is present both in the original network at the given threshold and the corresponding ideal network. A link is counted as ‘true negative’ when the link is absent both in the network at the given threshold and the corresponding ideal network. Whereas a link is defined as ‘false positive’ when it is present in the threshold-determined network but not in the corresponding true network, and ‘false negative’ when it is absent in the threshold-determined network but present the true network. An illustration of this scheme through a toy network is provided in Supplementary Fig. 10. Using these terms, we define accuracy α as the fraction of ‘true positive’ and ‘true negative’ links in an original threshold-determined network.

We find that with increasing correlation thresholds the network becomes closer to the expected true network as demonstrated by increasing values of accuracy for both processing-stage sample type and country of origin Fig. 3d (Spearman correlation network; Pearson correlation case in Supplementary Fig. 11). Further, in the region of low correlation threshold the character of the network is closer to that of the expected true network for the processing-stage sample type attribute, and in the region of higher correlation threshold the character of the network is closer to that of the expected true network for country-of-origin attribute. This result is in agreement with the previous results with formation of processing-stage sample type clusters at lower and intermediate correlation thresholds and of country-based clusters at high correlation thresholds.

In a previous investigation we discussed the origin country classification of cocoa bean samples based on linear discriminant analysis (LDA) applied to LC-MS data^43^. We showed that the classification strength can be substantially enhanced by applying an additional filtering step, called Gaussian feature stability^43^. Our network-based analysis now offers a mechanistic explanation for this statistical observation: Violation of the Gaussian feature stability criterion essentially means that for a set of samples from the same country, intensities of an LC-MS peak do not vary around a reliably defined average (e.g., due to outliers). In the network-based analysis presented here, such violations would be translated into reductions in sample-to-sample correlations. In this way, sample-based selection for very high correlations matches the compound-based selection due to Gaussian feature stability.

It is noteworthy that, in contrast to the LDA from Kumar et al.^43^, the network-based analysis presented here is *not* an example of ‘supervised learning’, but rather ‘unsupervised learning’, as its underlying classification task does not require a-priori information on the sample-to-country associations.

## Discussion

It is well known that the analysis of statistical associations in large-scale datasets is a useful strategy in food fingerprinting and food quality assessment. An example is the analysis of the statistical association of cheese microbiomes with volatile compounds presented in^44^, which furthermore shows the relevance of a detailed characterization of these microbial communities. In a recent overview^45^, Barabási et al. have outlined the tremendous potential of network science for food research. Here we introduce a network-based approach for quality control in cocoa research by classifying cocoa bean samples based on their high-throughput LC-MS profiles.

Classification of cocoa samples on the basis of their country of origin has been found challenging with limited success obtained in cases with the number of countries being few or the origin being on continental scale. Differences in unfermented and fermented samples can be easily seen by simply finding the Spearman correlation between the cocoa samples using their LC-MS profiles (cf. Figure 2c). The liquor samples are closer to the fermented samples. However, differentiation on the level of country of origin is only revealed upon further analysis. Evaluating correlation networks as a function of the discretization threshold, we find that differentiation of cocoa samples on the level of country of origin is on a more subtle level than their differentiation on the basis of processing-stage sample types.

**Fig. 4:**
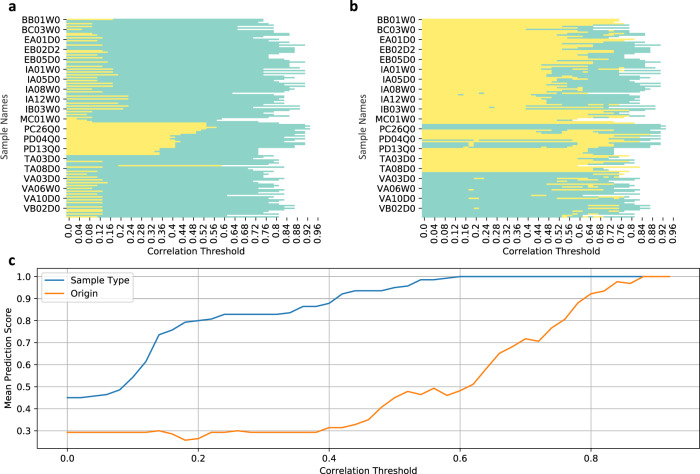
Simple majority vote model to infer sample-type or origin of a node/sample. (**a**) Sample-type. Prediction result for inference of sample-type of all nodes (vertical axis) at continuously increasing correlation thresholds (horizontal axis): green indicates correct prediction, yellow indicates false prediction. (**b**) Origin. Prediction result for inference of country of origin of all nodes. *Note*: (1) Only few sample names (not all) are shown on the vertical axis to avoid clutter, however all samples are represented in the heatmaps. (2) At high correlation thresholds corresponding networks become sparse thus loosing edges/nodes, hence, sample-type/origin inference for some nodes may not be possible. These are shown by white portion in the heatmaps. (**c**) Mean prediction score of sample-type and country of origin as a function of increasing correlation thresholds. It is evident that, on average, sample-type can be predicted correctly at mid and higher correlation thresholds, while origin is correctly predicted at higher correlation threshold regions.

How can the findings presented here be put to use for the purpose of cocoa bean fingerprinting? Let us assume we have a new sample, where the fermentation status and the origin country are not known, but where the LC-MS profile is known. By running the same analysis again, but including this sample, we can evaluate the new sample’s position in the correlation networks at different thresholds. A majority vote among the new sample’s neighbors in a network obtained at an intermediate threshold will reveal the new sample’s fermentation status, while a corresponding majority vote at high threshold will reveal its origin country. Should only a smaller database of already classified samples be available than the one analyzed here, the majority vote can be extended to next-to-nearest neighbors, in order to enhance statistics. The result of a majority vote model to infer a node’s sample-type or origin at various correlation threshold is shown in Fig. 4. Per expectations, sample-type predictions are often correctly made at mid and higher correlation thresholds, while origin predictions are often correctly made at higher correlation thresholds.

Our study takes into consideration two factors on which cocoa samples may primarily differ: processing-stage and country of origin. However, it is worth noting these are not the only governing factors that affects similarity of cocoa samples. Many other factors such as variety of cocoa hybrid, soil, climate, terrain, harvesting season, farming practices etc. also have significant effects^4,46–49^. It would be interesting to consider some of these factors and see, in what range of correlation threshold these effects start to matter and contribute to a modular organization of the corresponding correlation networks.

Furthermore, we intend to study the role of specific classes of compounds in clustering of samples at different correlation thresholds. Which compounds serve as fermentation separators, which as country-of-origin separators? Does the clustering depend on the nature of the compounds (e.g., polyphenols, carbohydrates, peptides, primary or secondary metabolites)? Can the tools of network science answer these question, or what augmentations must be brought into network science framework to achieve success at such questions?

But to begin with, this work sets out a network-based framework that besides providing a new perspective on chemical similarity of cocoa bean samples, can be a complement to the traditional chemoinformatics approaches in food research in general.

## Methods

### Country of origin details

The LC-MS data set we use here has a total of 140 samples (positive ion mode). The samples have been gathered and their LC-MS profiling done over a range of about four years. These samples can be grouped into three sample types (Unfermented, Fermented and Liquors) and eight origins (Brazil, Cameroon, Ecuador, Ghana, Indonesia, Ivory Coast, Malaysia, and Tanzania) (Fig. 2a).

### Data pre-processing and cleaning

The data generation, cleaning, standardization and organization has been discussed in an earlier work^43^. Briefly, LC-MS data of all the samples was processed using MZMine^50^ giving peak area list and corresponding *m/z ratio* and *retention times*. The detected compounds are assigned names/chemical formula on the basis of four ionization states ([M + H], [M + 2H], [M + 3H], [2 M + H]) when possible, else the compound was named as ‘Unknown_’ suffixed with the *m/z* value, e.g., Unknown_865.1927. The sum of peak area values belonging to each sample was normalized to 100, so the peak area in the sample represents relative percentage amount of compound in the LC-MS profile. Henceforth, we refer to the percentage normalized peak area as the peak areas or concentration of compounds. The samples were then put in an excel file, where each row represents a sample, and the column contain information about the sample-type, origin and peak areas of various compounds (≈7000) sorted in descending order by their mean peak are across all the samples (140). This excel table is provided in *supporting_data.xlsx* under sheet named *lcms_data*. We additionally clarify that our results remain qualitatively the same for a good range (top/first 1000 to 7000) of compounds and does not alter the conclusions drawn out in this manuscript. Therefore, additional data cleaning, e.g., keeping only compounds present in at least a certain percentage of samples, is not required for purposes of this manuscript. Nevertheless, for tasks that require more computation/time (e.g., obtaining statistics for a series of correlation thresholds) we recommend using first 1000 compounds without sacrificing on the generality of inferences. We ourselves follow the same strategy.

### Correlation measures: Spearman and pearson correlation

A typical LC-MS profile contains information about thousands of compounds present in a given sample defined by their retention time and associated *m/z* values^51^. The preprocessed and cleaned LC-MS data can be represented as a matrix L with entries $l_i^\alpha$. The upper index α represents the sample and lower index *i* represents the compound. Thus, the scalar quantity $l_i^\alpha$ represents the concentration of *i*^th^ compound in the α^th^ LC-MS sample. Correspondingly, *l*^α^ is a vector which represents the LC-MS profile of sample α. The Pearson correlation between two LC-MS samples, say α and β with corresponding profiles *l*^α^ and *l*^β^, can be denoted as *r*_αβ_. It is calculated as

1 $$\begin{array}{*{20}{c}} {r_{\alpha \beta } = \frac{{{{{{{\mathrm{cov}}}}}}\left( {l^\alpha\!,\,l^\beta } \right)}}{{\sigma _{l^\alpha }\sigma _{l^\beta }}}} \end{array}$$

where cov(*l*^α^, *l*^β^) represents the covariance between the LC-MS profiles of samples α and β, while $\sigma _{l^\alpha }$ and $\sigma _{l^\beta }$ represent the standard deviation in the LC-MS profiles *l*^α^ and *l*^β^, respectively. The Spearman correlation can be defined as the Pearson correlation between the ranks of the original variables (i.e., *l*^α^ and *l*^β^). The ranked variables $\tilde l^\alpha$ and $\tilde l^\beta$, are obtained from the original variables *l*^α^ and *l*^β^ by sorting them from lowest to highest and substituting the values by the position in the sorted list (i.e., the rank of the values). Formally, the Spearman correlation coefficient is thus calculated as

2 $$\begin{array}{*{20}{c}} {\tilde r_{\alpha \beta } = \frac{{{{{{{\mathrm{cov}}}}}}\left( {\tilde l^\alpha\!,\,\tilde l^\beta } \right)}}{{\sigma _{\tilde l^\alpha }\sigma _{\tilde l^\beta }}}} \end{array}$$

The Spearman and Pearson correlations across all pairs of LC-MS samples can be written in the form of matrices, ${{{\tilde{\mathrm R}}}}$ and R, whose entries denoted by $\tilde r_{\alpha \beta }$ and $r_{\alpha \beta }$, respectively.

### Network production and visualization

A network is defined through two sets of entities: nodes (*N*) and edges (*E*). The nodes denote the objects which are related to each other in some way, and the edge represent the relation between the nodes. In a correlation network, an edge represents the correlation between two nodes. In our correlation network, the nodes represent the different LC-MS samples of cocoa or its products sourced from different origins, and the edge between the nodes represent the correlation between the LC-MS samples. Spearman and Pearson correlation analysis, and network generation/transformation were done using the Python programming language. Network visualization has been done in Cytoscape^52^. For layout of the network either of the following two variants of spring layout, which were available in Cytoscape itself, were used: (a) Edge-weighted Spring Embedded Layout^53^, (b) Compound Spring Embedder (CoSE)^54^. These layouts consider the weight of the edge (in our case the Spearman or Pearson correlation coefficient) between nodes, so that the nodes with higher weight of edges (correlations) between them are placed closer together. Figure 2b shows the correlation network obtained by using all correlations (0 to 1) between all LC-MS samples and visualized with edge-weighted spring layout. Metadata about the LC-MS samples, such as country, and processing-stage sample type (unfermented, fermented, or liquor) has been represented through color and shape of nodes, respectively. The network shown in Fig. 2b is the correlation network made using Spearman correlation and has 140 nodes and 6833 edges, i.e., 140 cocoa LC-MS samples and 6833 correlations ($\tilde r$ > 0) between the nodes. The network made using Pearson correlation is shown in the Supplementary Fig. 1. The label of the node represents the internal LC-MS id. The strength of correlation is represented by the color of the edge between the nodes, yellow representing low correlation and violet representing high correlation. The spatial placement of nodes in Fig. 2b, and all of the following networks, is done through variants of spring layout algorithms in Cytoscape^52^ which places the nodes with higher correlation closer together.

### Null model network or control network

A null model network is created by randomizing the weights (correlations) of edges in the original correlation network. It is important to note that the null model network so obtained has the same correlation distribution as that of the original correlation network because the set of correlations in the network remains unchanged, only the correlations between nodes is randomized. An ensemble of 100 such null model networks were generated. The reported statistics about a studied property on the null model networks is obtained by making calculations over this ensemble and then reporting the mean and standard deviation of the studied property. Higher the difference in the studied property between the original network and null network ensemble, higher the significance of the observed property in the original network.

### Reporting Summary

Further information on research design is available in the Nature Research Reporting Summary linked to this article.

## Supplementary information

Supplementary Information (tables and figures).

Supplementary_Movie_Spearman_correlation_sample-type

Supplementary_Movie_Spearman_correlation_origin

Dataset 1

REPORTING SUMMARY

## Data Availability

The data used in this study is available in this published article LC-MS_and_network.xlsx.

## Code availability

The code is available from the authors upon request.
