# Supplementary material for: Cocoa bean fingerprinting via correlation networks
Source: NPJ Sci Food. 2022 Jan 24;6:5. doi: 10.1038/s41538-021-00120-4 (PMC8786884; doi:10.1038/s41538-021-00120-4)
Supplement: Supplementary file 1 — Supplementary Information (tables and figures). [file 41538_2021_120_MOESM1_ESM.pdf]

Supplementary Information

Cocoa bean fingerprinting via correlation networks

Santhust Kumar,[1*] Roy N. D'Souza,[1] Marcello Corno,[2] Matthias S. Ullrich,[1] Nikolai Kuhnert[1], and Marc-Thorsten Hütt[1*]

[1]Department of Life Sciences and Chemistry, Jacobs University Bremen, Campus Ring 1, 28759 Bremen, Germany

[2]Barry Callebaut AG, Westpark, Pfingstweidstrasse 60, Zurich 8005, Switzerland

*Correspondence to:

Dr. Santhust Kumar (s.santhust@jacobs-university.de)

Prof. Dr. Marc-Thorsten Hütt (m.huett@jacobs-university.de)

Department of Life Sciences & Chemistry

Jacobs University Bremen gGmbH

Campus Ring 1

28759 Bremen, Germany

Table of Contents

1 *Sample distribution table* *3*

2 *Pearson correlation* *4*

3 *Networks at low and intermediate correlation* *5*

3.1 Spearman correlation 5

3.2 Pearson correlation........ 6

4 *Country enriched modules at high correlation threshold* *7*

4.1 Spearman correlation (countries colored) 7

4.2 Spearman correlation (Processing stage colored) 8

4.3 Pearson correlation........ 9

4.4 Number of nodes and edges as a function of correlation threshold in networks using Spearman and Pearson correlation........ 11

5 *Similarity of nodes connected by edges in networks made using Pearson correlation* *12*

6 *Accuracy of links in thresholded correlation networks* *13*

6.1 Toy network illustrating accuracy concept 13

6.2 Accuracy of link in correlation network made using Pearson correlation as a function of correlation thresholds 14

7 *Videos* *15*

1 Sample distribution table

	Brazil	Cameroon	Ecuador	Ghana	Indonesia	Ivory Cost	Malaysia	Tanzania	All
**Unfermented**	4	3	8	0	14	16	6	3	**54**
**Fermented**	4	3	12	0	16	16	3	9	**63**
**Liquor**	0	6	3	5	0	9	0	0	**23**
Total	**8**	**12**	**23**	**5**	**30**	**41**	**9**	**12**	**140**

Supplementary Table 1 The LCMS data set can be grouped on twin axes: sample-type and origin. There are 3 sample-types: Unfermented, Fermented and Liquors, and there are 8 origins (Brazil, Cameroon, Ecuador, Ghana, Indonesia, Ivory Coast, Malaysia and Tanzania).

2 Pearson correlation

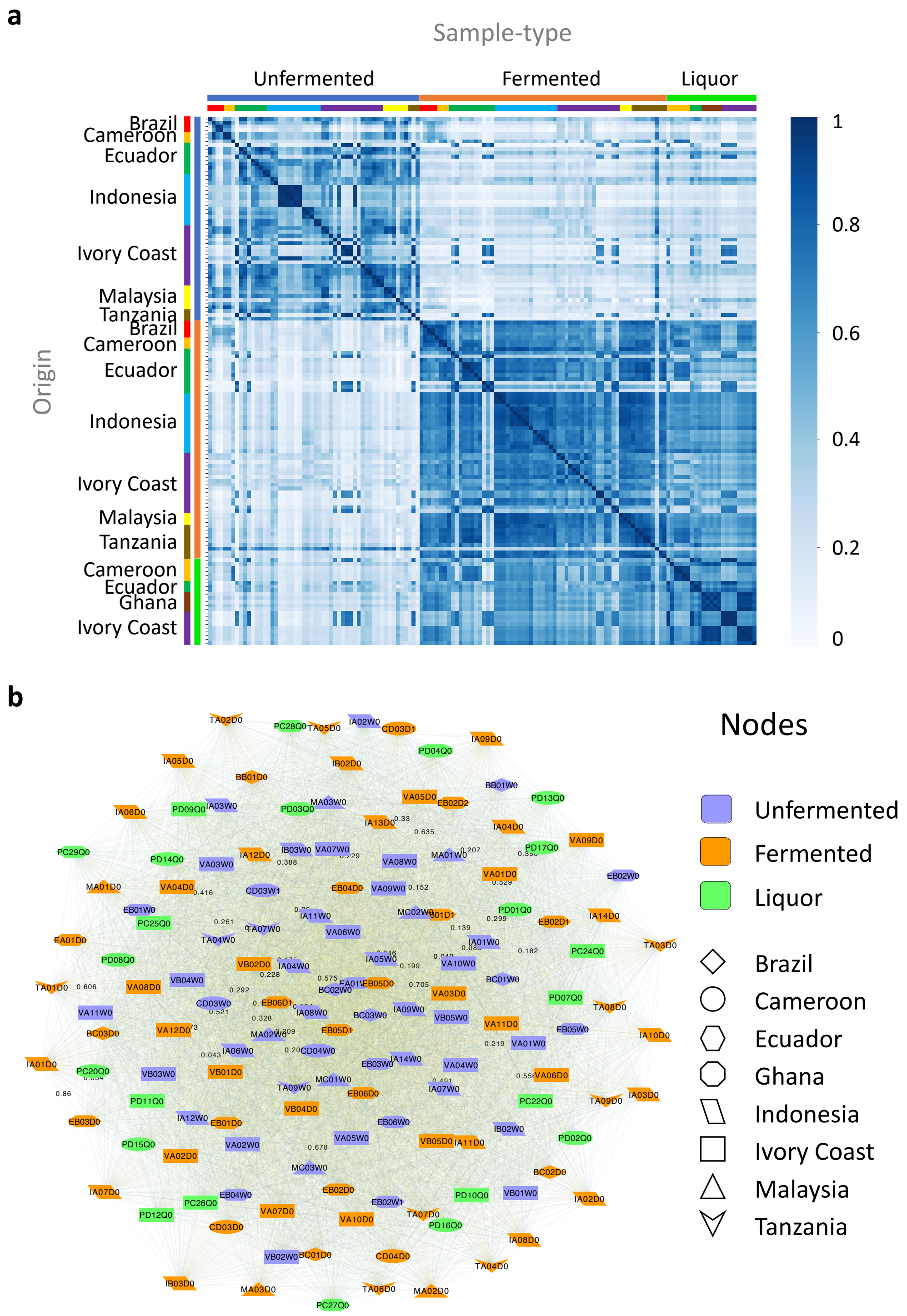

Supplementary Figure 1 Correlation between cocoa samples. Similar to Figure 2b,c in main text but using Pearson correlation instead of Spearman correlation. **(a)** Correlation heatmap**.** The distinction between the Fermented and Liquor samples is lesser compared to that brought out using Spearman correlation. Unfermented samples are easily distinguishable from the Fermented and Liquor samples. **(b)** Correlation Network. The correlations are computed using Person correlation coefficient.

3 Networks at low and intermediate correlation

3.1 Spearman correlation

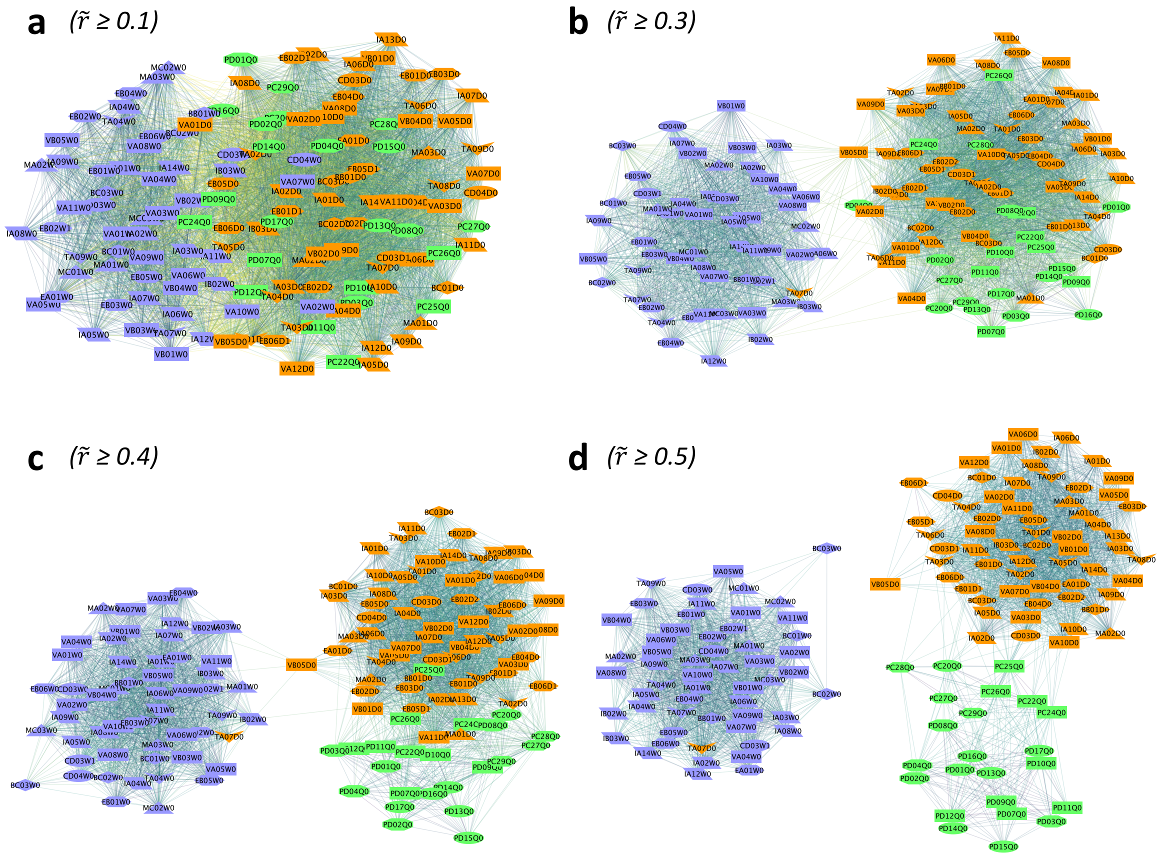

Supplementary Figure 2 Processing-stage modules in low and intermediate threshold correlation networks. Similar to Figure 3a in the main text but with labelled nodes. The figure reveals modules of samples belonging to the same cocoa processing-stage in a typical cocoa processing pipeline. **(a)** Network of LC-MS samples at a correlation threshold of 0.1 revealing separation of unfermented, fermented and liquor cluster. **(b, c)** Correlation thresholds 0.3 and 0.4. The separation between different processing-stage sample types improves. **(d)** Correlation threshold 0.5. Three groups of unfermented, fermented and liquor samples are clearly separated. The figure follows same legend as of Supplementary Supplementary Figure **1**. See Supplementary Video for a movie on evolving network as the correlation threshold is progressively increased.

3.2 Pearson correlation

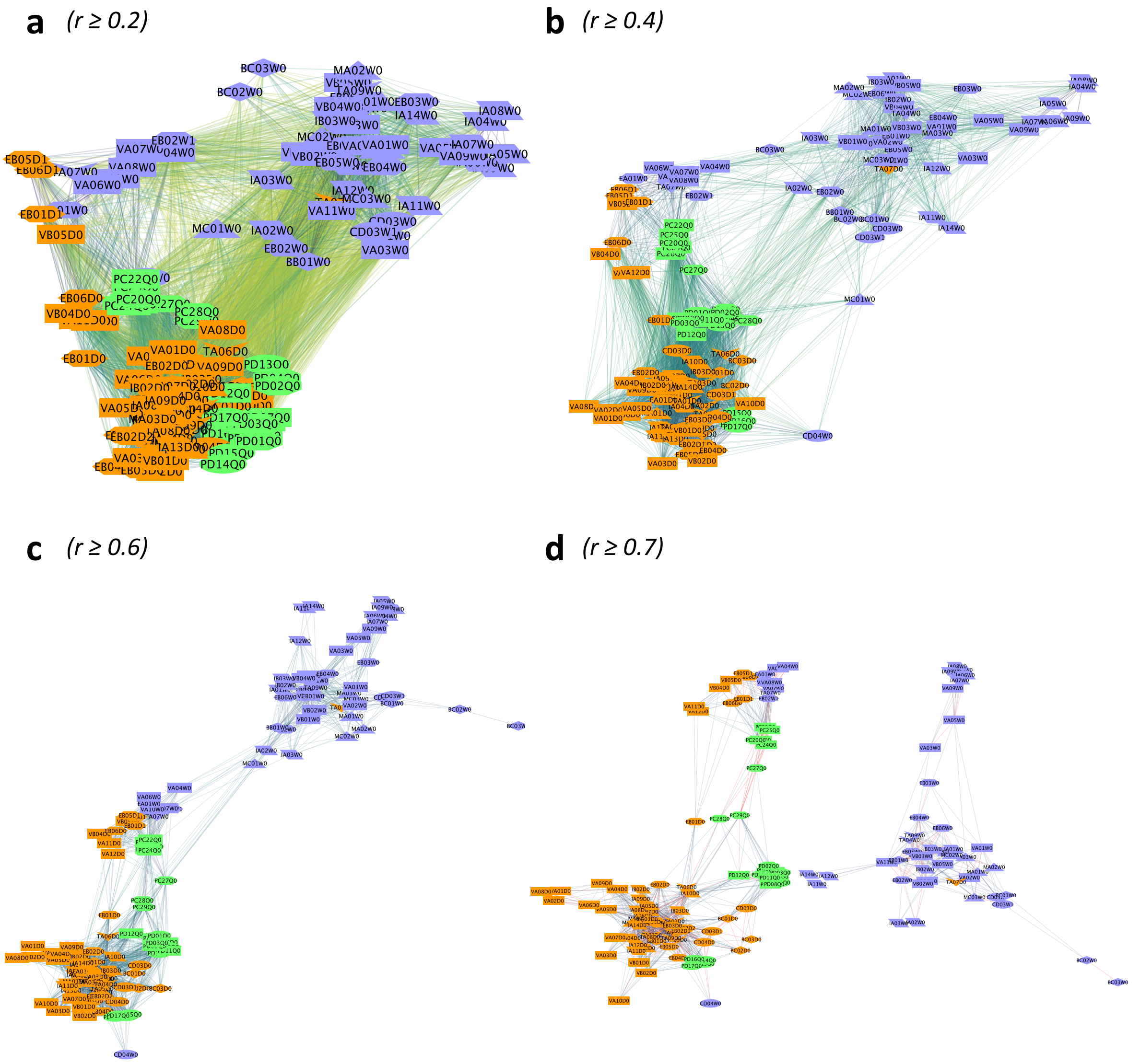

Supplementary Figure 3 Correlation Network at different correlation thresholds. Similar to Supplementary Supplementary Figure **2** above, but the Pearson correlations are used to make the network. The revelation of substructure of the network showing Unfermented, Fermented and Liquor occurs at different threshold. Substructures are also revealed.

4 Country enriched modules at high correlation threshold

4.1 Spearman correlation (countries colored)

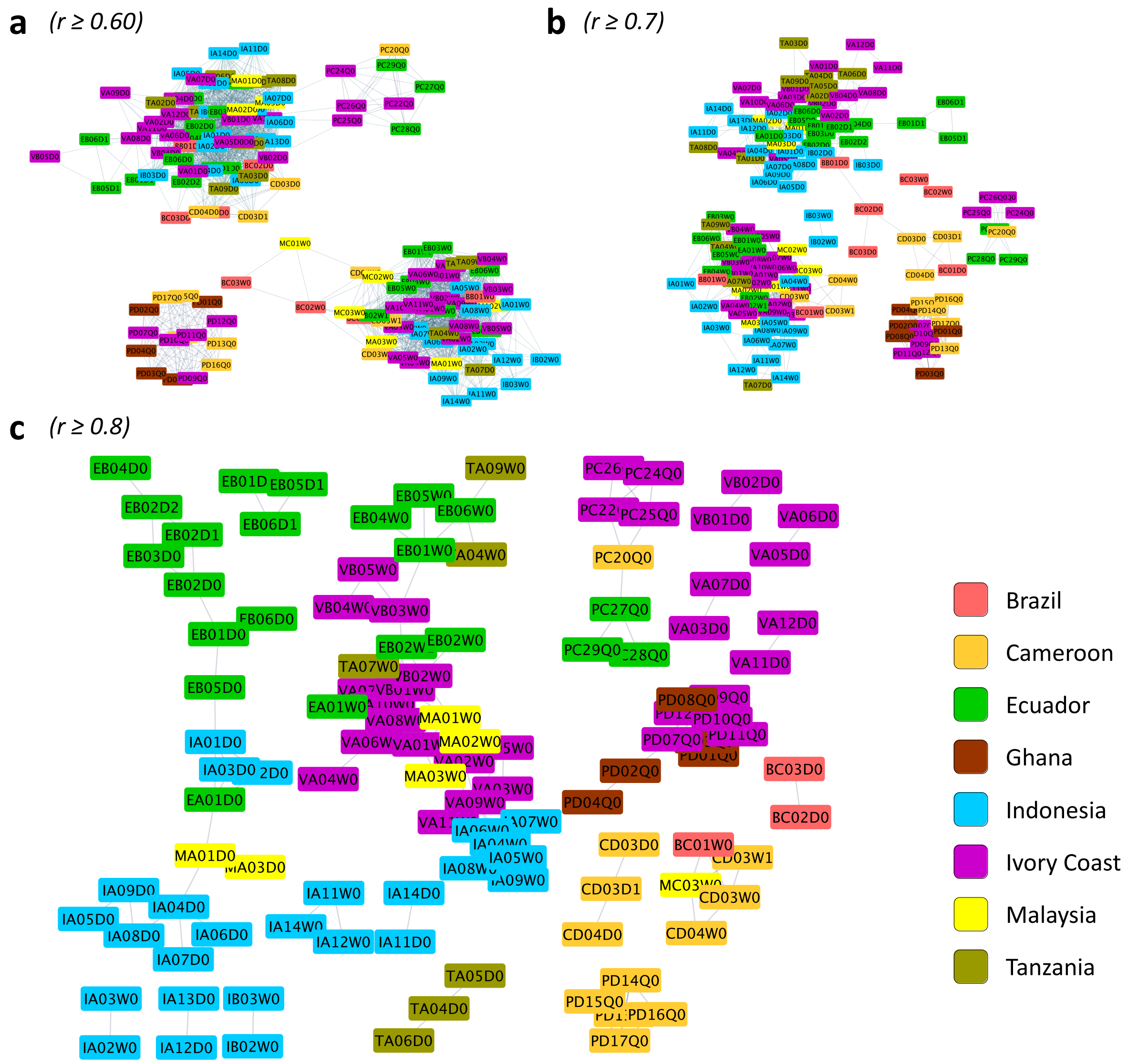

Supplementary Figure 4 Country modules. Similar to Figure 3b in main text but with labels of the nodes shown. The structure of correlation network of cocoa samples based on their LCMS profile at correlation thresholds of 0.6, 0.7 and 0.8. At these correlation thresholds, several modules with nodes belonging to the same country of origin are revealed. For a quick and better comprehension and unlike the legend of earlier correlation networks, in this figure different countries are represented through a different color (see Supplementary Supplementary Figure **5** ahead for nodes colored according to processing stage, i.e., fermented/unfermented/liquor stages). See Supplementary Video for a movie on evolving network as the correlation threshold is progressively increased.

4.2 Spearman correlation (Processing stage colored)

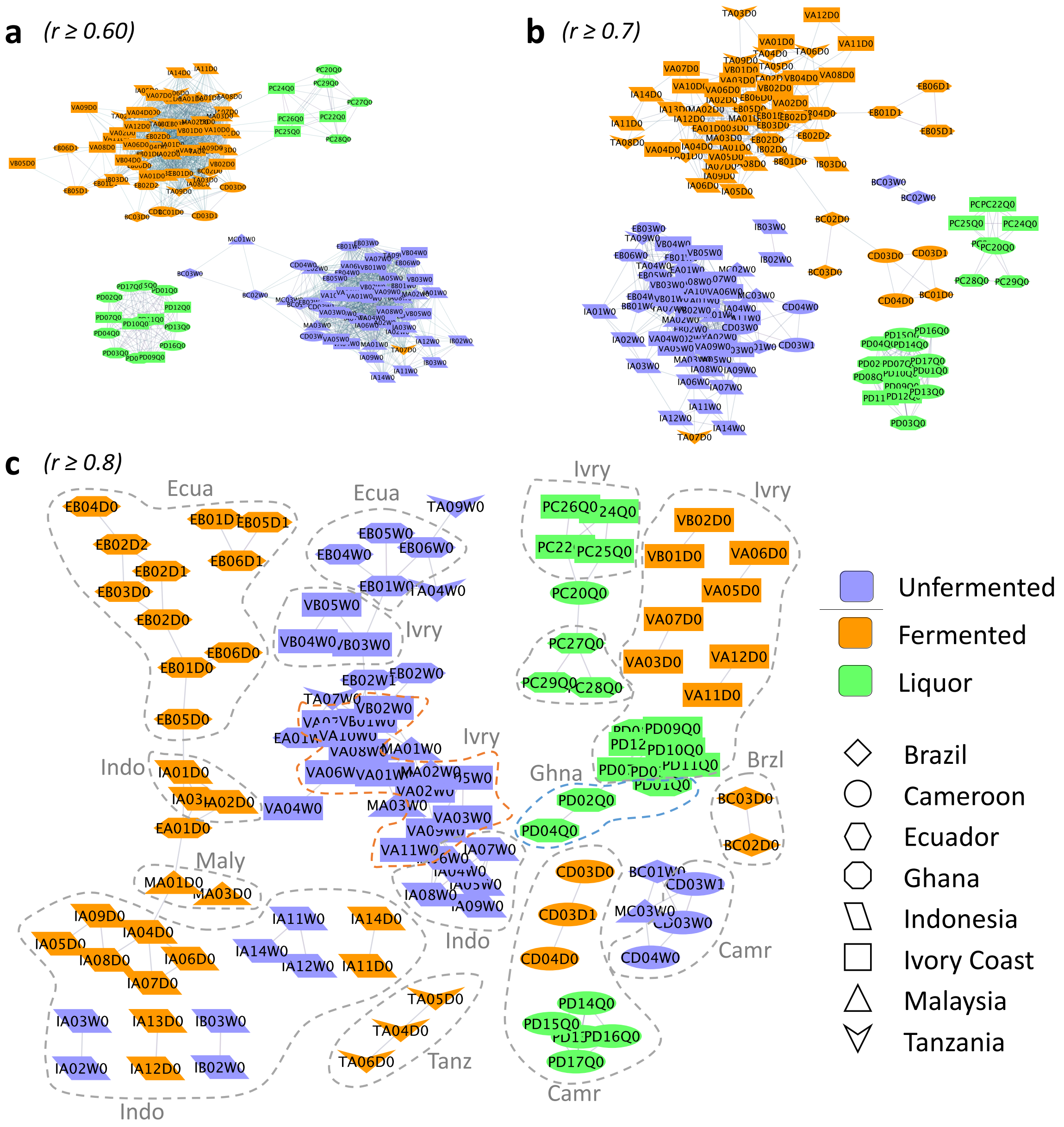

Supplementary Figure 5 Country modules revelated at higher correlations: Similar to Supplementary Supplementary Figure **4** above, but with node color representing sample-type and node shape representing countries of origin of cocoa sample. For easy comprehension of revealed grouping on the basis of origin, nodes belonging to the same country have been demarcated with dotted lines, and labelled with country legend—*Brzl*: Brazil, *Camr*: Cameroon, *Ecua*: Ecuador, *Ghna*: Ghana, *Indo*: Indonesia, *Ivry*: Ivory Coast, *Maly*: Malaysia; *Tanz*: Tanzania. We would like to emphasize, for the sake of clarity and avoiding potential confusion, that dashed lines around the clusters should not be taken to assume that various statistics in the article are generated over cluster of nodes. The color and shape of the dotted lines are only for the purpose of clear comprehension; it does not have any further connotation.

4.3 Pearson correlation

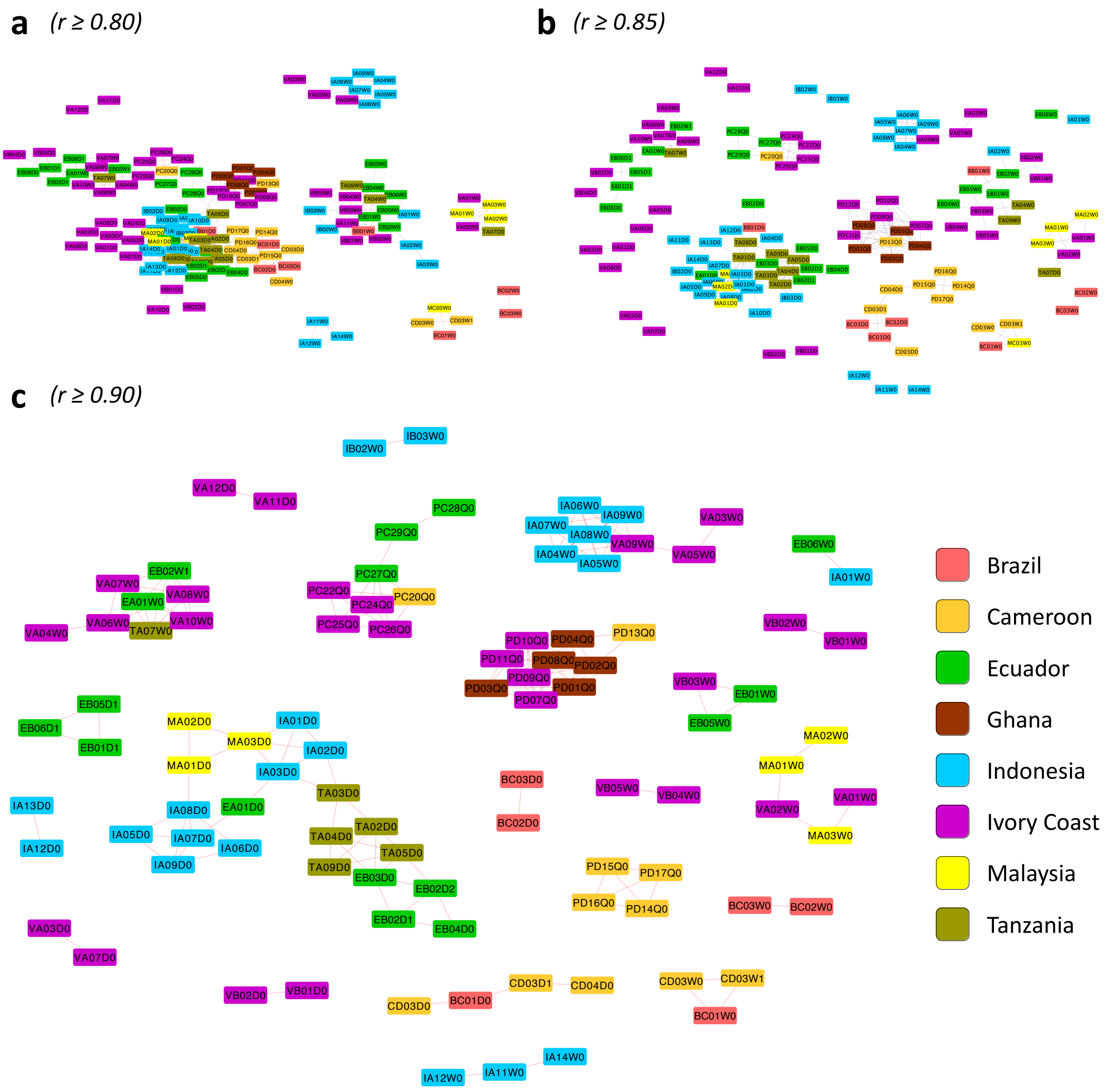

Supplementary Figure S6 Country modules revelated at higher correlations: Similar to Figure 3b in main text and Supplementary Supplementary Figure **4** above, but with Pearson correlation instead of Spearman correlation.

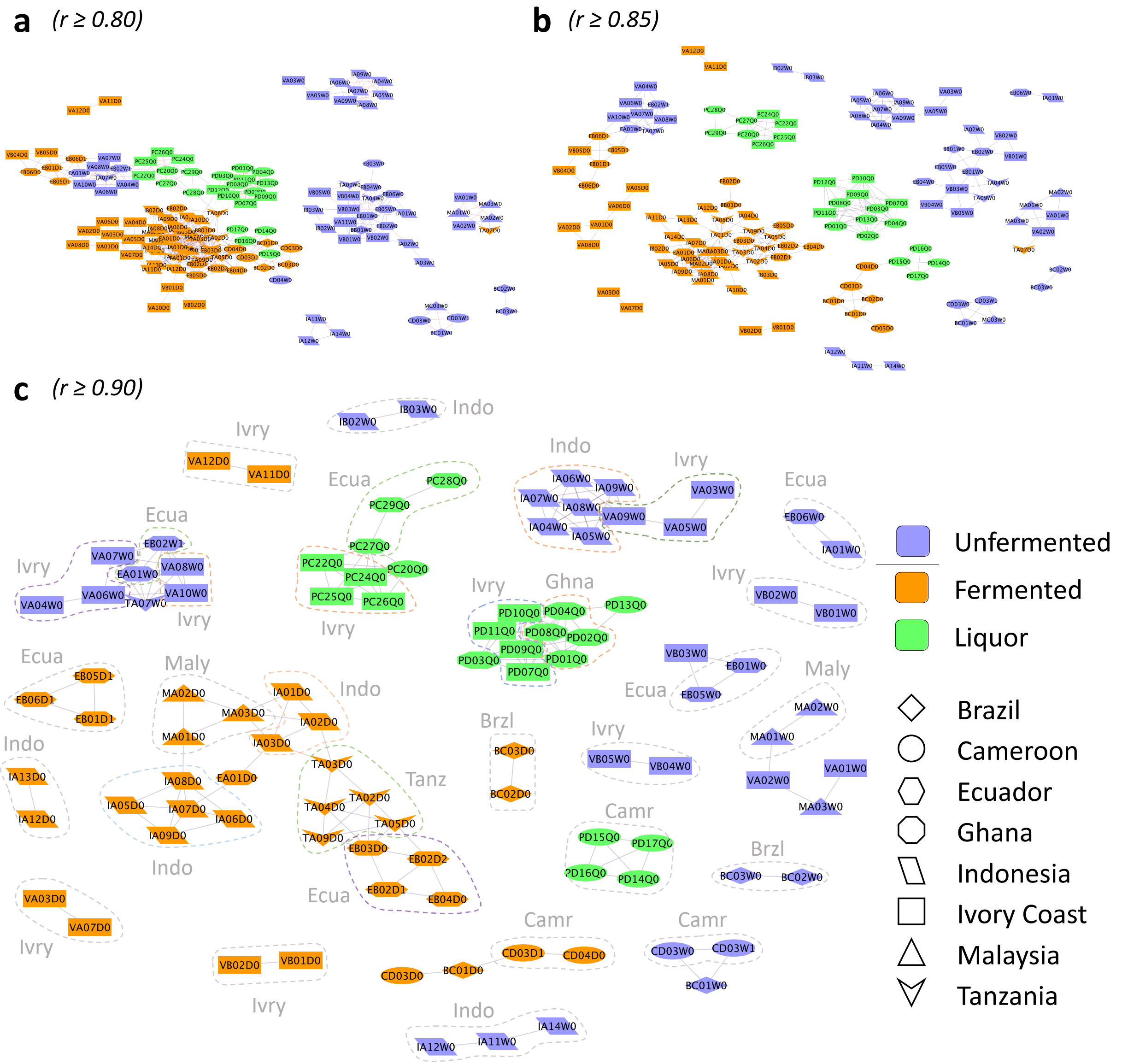

Supplementary Figure S7 Country modules. Similar to Supplementary Supplementary Figure **5** above but using Pearson correlation coefficient instead of Spearman correlation, and with node color representing sample-type and node shape representing countries of origin of cocoa sample. For easy comprehension of revealed grouping on the basis of origin, nodes belonging to the same country have been demarcated with dotted lines, and labelled with country legend—*Brzl*: Brazil, *Camr*: Cameroon, *Ecua*: Ecuador, *Ghna*: Ghana, *Indo*: Indonesia, *Ivry*: Ivory Coast, *Maly*: Malaysia; *Tanz*: Tanzania. The color and shape of the dotted lines are only for the purpose of clear comprehension; it does not have any further connotation. The electronic version of the image can be zoomed in for details.

4.4 Number of nodes and edges as a function of correlation threshold in networks using Spearman and Pearson correlation

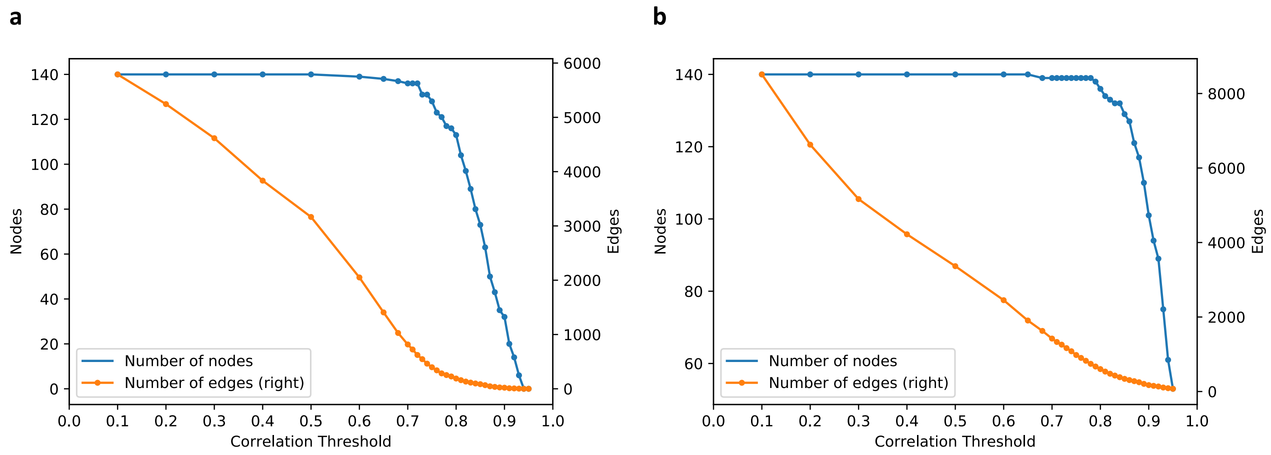

Supplementary Figure S8 Number of Edges and nodes at different correlation thresholds in correlation network made using Spearman correlations. **(a)** Nodes and edges in network made using Spearman correlations. As the correlation threshold in increased, number of edges in the resulting networks drops sharply. The number of nodes remains almost constant for a wide range of the correlation threshold (0, 0.6), changes slowly in range (0.6, 0.75), and afterwards witness a sharp fall. **(b)** Nodes and edges in network made using Pearson correlations. The number of nodes remains almost constant for a wide range of the correlation threshold (0, 0.7), changes slowly in range (0.7, 0.8), and afterwards witness a sharp fall.

5 Similarity of nodes connected by edges in networks made using Pearson correlation

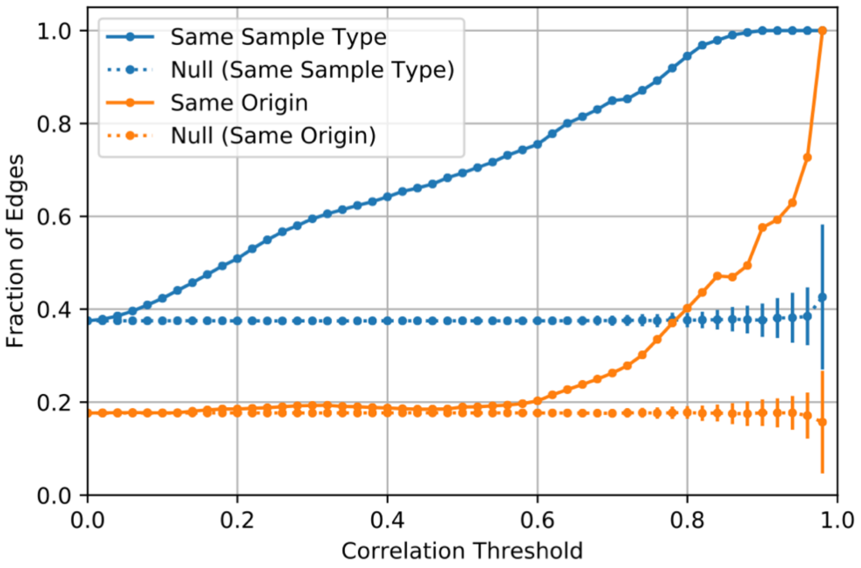

Supplementary Figure S9 Connected nodes' similarity in network made using Pearson correlation coefficient. Similar to Figure 3c in the main text, but in the correlation network made using the Pearson correlation instead of Spearman correlation. Qualitatively, the behavior is same in both cases, i.e., Spearman correlation network and Pearson correlation network. In the latter case, i.e., Pearson correlation network, same similarity values are reached at higher correlation thresholds. In both cases, sample-type similarity value remains high, and origin similarity catches up at high correlation thresholds.

6 Accuracy of links in thresholded correlation networks

6.1 Toy network illustrating accuracy concept

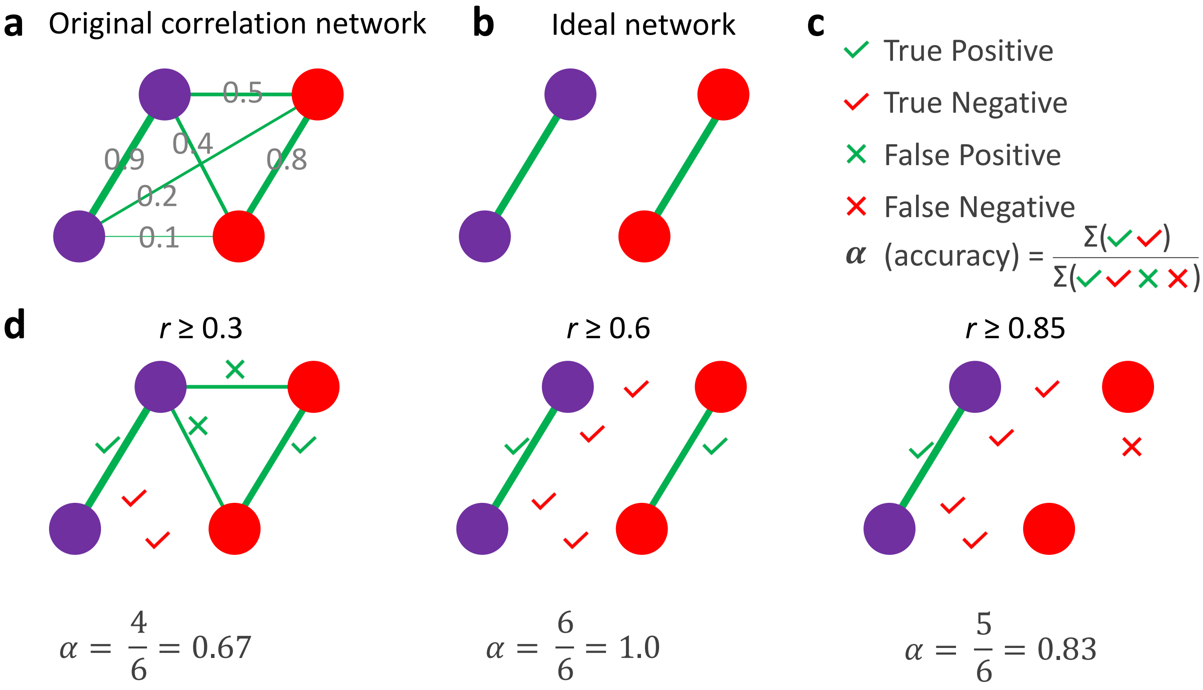

Supplementary Figure 10 Toy example illustrating calculation of accuracy of links using original and expected ideal network at different correlation thresholds. Same color of the node may be taken as representing one of the either attribute of cocoa samples: sample type or country of origin. **(a)** The original correlation network obtained after finding correlation between cocoa samples. The correlations between nodes are mentioned on respective links/edges. **(b)** The expected ideal network: it has links only between same color nodes (same attribute: sample-type or same origin). **(c)** Legend for the link type in threshold-fixed networks. True positive: link present in both threshold-fixed and ideal network; true negative: link absent in both threshold-fixed and ideal network; false positive: link present in threshold-fixed network but absent in ideal network; and false negative: link absent in threshold-fixed network but present in ideal network. Accuracy: fraction of true positive and true negative links in the threshold-fixed network. **(d)** Example networks at different [correlation] thresholds and accuracy of the links, or alternatively, closeness of the original threshold-fixed network to the expected ideal network.

6.2 Accuracy of link in correlation network made using Pearson correlation as a function of correlation thresholds

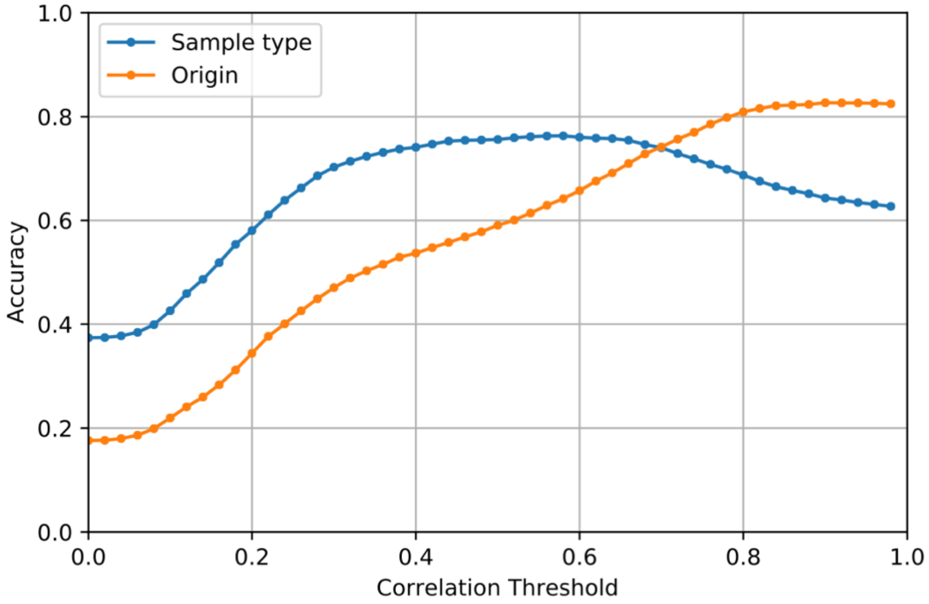

Supplementary Figure 11 Accuracy of links in threshold-fixed correlation networks, or closeness of a threshold-fixed correlation network to expected ideal network based on sample type or origin attributes of cocoa samples. Similar to in Figure 3d in main text, but in the correlation network made using Pearson correlation instead of Spearman correlations. Compared to the case of Spearman correlation network in Figure 3d in main text, here the plateau for sample-type remains lower than 0.8. However, in both cases, the result qualitatively remains the same: at lower threshold the correlation networks are closer in character to the sample type feature of samples, while at higher thresholds the correlation networks are closer in character to the origin feature of samples.

7 Videos

Supplementary Video 1 Animated separation of sample modules based upon sample type. movie_spearmanCorrelation_sampleType.mp4 shows how the network of samples separates into different modules (unfermented samples, fermented samples, liquor samples) as the correlation threshold is gradually increased.

Supplementary Video 2 Animated separation of sample modules based upon origin. movie_spearmanCorrelation_origin.mp4 shows how the network of samples separates into different origin-based modules at higher correlation thresholds.
